# High prevalence and risk factors of fecal carriage of CTX-M type extended-spectrum beta-lactamase-producing Enterobacteriaceae from healthy rural residents of Taian, China

**DOI:** 10.3389/fmicb.2015.00239

**Published:** 2015-03-27

**Authors:** Hongna Zhang, Yufa Zhou, Shuyuan Guo, Weishan Chang

**Affiliations:** ^1^College of Animal Science and Technology, Shandong Agricultural UniversityTaian, China; ^2^College of Animal Science and Technology, Shanxi Agricultural UniversityTaigu, China; ^3^Animal Husbandry Bureau of DaiyueTaian, China

**Keywords:** CTX-M type extended-spectrum beta-lactamase (ESBL), healthy rural residents, Enterobacteriaceae, risk factors, Taian

## Abstract

The study was carried out to understand the prevalence of CTX-M type extended-spectrum beta-lactamase (ESBL)-harboring Enterobacteriaceae and to analyze risk factors related with fecal carriage in healthy rural residents in Taian, China. A total of 620 stool samples were collected from rural residents. The ESBL-positive Enterobacteriaceae was screened using ChromID ESBL agar, and then further confirmed by double-disk diffusion. The CTX-M genes were determined using polymerase chain reaction. The risk factors associated with fecal carriage of CTX-M-positive isolates were analyzed using the standard statistic methods. 458 isolates carrying CTX-M gene (458/620, 73.9%) were obtained from different individuals, and the most dominant genotype was CTX-M-9 group (303/458, 66.2%). The dominant species were *Escherichia coli* (*E. coli*; 403/458, 88.0%) and* Klebsiella pneumoniae* (*K. pneumoniae*; 26/458, 5.7%) among the isolates carrying CTX-M genes. All the CTX-M producers were resistant to ampicillin, cefazolin, cefuroxime, and ceftriaxone, but were all susceptible to biapenem, imipenem, and meropenem. The results of multivariate logistic regression model identified the enrollment in formal education (OR 2.321; 95% CI 1.302–3.768; *P*= 0.039), the hospitalization history within the last 6 months (OR 1.753; 95% CI 1.127–2.584; *P*= 0.031) and the antibiotics use within the last 6 months (OR 1.892; 95% CI 1.242–2.903; *P*= 0.034). The three variables were significantly associated with carriage of CTX-M ESBL producers (*x*^2^ = 21.21; *df* = 3; *P*< 0.001). The prevalence of fecal carriage of CTX-M ESBL-producing Enterobacteriaceae among healthy rural humans in Taian was high, and the recent antibiotic use and hospitalization history may be the important contributors.

## Introduction

The main resistance mechanism of Enterobacteriaceae against beta-lactam antibiotics is the production of extended-spectrum beta-lactamases (ESBL). The enzyme can hydrolyse penicillins, cephalosporins, and aztreonam, but they can be inhibited by clavulanic acid, sulbactam, and tazobactam (Bush et al., 1995). The global epidemic ESBL mainly includes SHV, TEM, and CTX-M types. During the past decade, the CTX-M type has been recognized as the predominant beta-lactamase in Enterobacteriaceae throughout the world (Bell et al., 2002; Livermore et al., 2007; Doi et al., 2013; Zhang et al., 2014).

A number of studies have shown that CTX-M type ESBL-producing Enterobacteriaceae not only cause hospital-acquired infections but also are the main cause of community-onset bloodstream infections. In addition, The ESBLs are plasmid-encoded enzymes, and the plasmids have the potential to transfer between Enterobacteriaceae, which further aggravate the public concerns (Bingen et al., 1993; Ben-Ami et al., 2006; Rodríguez-Baño et al., 2006; Pitout and Laupland, 2008). In China, the prevalence of ESBL-producing Enterobacteriaceae causing nosocomial infections in tertiary hospitals exhibited a high increase from less than 20.0 to 72.2% between 2000 and 2011 (Xiao et al., 2011, 2012). Another investigation of Chinese county hospitals carried out during 2010–2011 showed that the incidence of ESBL-producing Enterobacteriaceae causing community-onset infections varied from 30.2 to 57.0% in different regions (Zhang et al., 2014). It is noteworthy that the two nationwide investigations on ESBL-producing Enterobacteriaceae showed that the majority of ESBL-producing Enterobacteriaceae carried *bla*_CTX-M_ genes.

CTX-M type ESBL-producing Enterobacteriaceae has been no longer limited to community-onset or hospital-acquired infections. Fecal carriage of CTX-M type ESBL-producing Enterobacteriaceae from the healthy individuals has been noted in many regions across the world (Guimarães et al., 2009; Kader and Kamath, 2009; Vinué et al., 2009; Herindrainy et al., 2011; Janvier et al., 2011). But little information about the characterization of CTX-M type ESBL-producing Enterobacteriaceae isolated from healthy rural residents in Chinese villages is very little.

It is well-known that most Chinese people live in the rural areas, and meanwhile there are high prevalence of CTX-M ESBL-producing Enterobacteriaceae among hospital-acquired and community-onset infections. Therefore it is of utmost importance to investigate the prevalence of CTX-M ESBL-producing Enterobacteriaceae isolated from healthy rural humans in China and analyze its relatedness with risk factors.

## Materials and Methods

### Ethics Statement

This present study was approved by the Ethics Committee of Shandong Agricultural University. Written informed consent was obtained from each subject participating in the study.

### Sample Collection and Questionnaires

Sampling was performed between October, 2013 and February, 2014 in two counties (Xintai and Ningyang) of Taian, China. The participants were selected by random door-to-door sampling. A total of 650 people aged >18 years (Xintai:330; Ningyang: 320) were approached to participate in this study, of whom 30 humans refused (Xintai:10; Ningyang: 20).

Before the stool sampling, participants were interviewed and the related information was recorded. The contents of questionnaires included: age, gender, education, antibiotic usage in the previous 6 months, and admission to hospital in the previous 6 months.

### Isolation and Confirmation of ESBL-Producing Enterobacteriaceae

The fresh stool samples were collected by the subjects using a nylon flocked ESwab 480CE (Copan, Brescia, Italy), and then the swab was spread onto the ChromID ESBL agar (BioMerieux, Marcy l’Etoile, France). After the incubation at 37.8^∘^C for 24 h, the confirmation of ESBL phenotype was carried out by the disk diffusion method according to the recommendation of CLSI (2011). The positive isolates were further identified using conventional biochemical tests and the API 20E system (Sysmex-bioMerieux, Tokyo, Japan).

### Antimicrobial Susceptibility Testing

The disk diffusion method was used in this study to test the susceptibility of all the ESBL-producing Enterobacteriaceae to 12 commonly used antibiotics in clinical practices, including ampicillin, cefazolin, cefuroxime, ceftriaxione, cefoxitin, biapenem, imipenem, meropenem, piperacillin, gentamicin, amikacin, and fosfomycin (Chinese National Institute for the Control of Pharmaceutical and Biological Products). *Escherichia coli* (ATCC 25922) and *Klebsiella pneumoniae* (ATCC 700603) were used as the quality control strains in this study (CLSI, 2011).

### Identification of *Bla*_**CTX-M**_ Gene

The extraction of DNA was conducted by boiling suspensions of the ESBL-producing isolates, and then the identification of *bla*_CTX-M_ genes were performed by multiple polymerase chain reactions (PCR; Monstein et al., 2007). According to the previous reference, four primer sets were used to amplify group-specific* bla*_CTX-M_ genes (CTX-M-1, CTX-M-2, CTX-M-8 and CTX-M-9; Pitout et al., 2004).

### Statistical Analysis

Mann–Whitney *U* test and *x*^2^ test were used to compare the continuous data and the categorical data, respectively. The univariate and multivariate logistic regression was used to analyze the risk factors associated with the fecal carriage of ESBL-producing Enterobacteriaceae. The results were presented as OR with 95% CI. Significance was set at *P*< 0.05. All the data were analyzed by SPSS version 18.0 software (The Predictive Analytics Company, Chicago, IL, USA).

## Results

There were 320 and 300 stool samples collected in Xintai and Ningyang counties, respectively. The information about demographics and hospitalization history and antibiotics use within 6 months was recorded (**Table 1**). Better education and higher month income were found in the rural subjects from Xintai county.

**Table 1:** Characteristics of the study participants.

Characteristics	Locations		*p*-value^a^
	Xintai, *N* = 320	Ningyang, *N* = 300	
Age (years), median (range)	42 (20–80)	45 (20–80)	0.565
Female gender	159 (49.7%)	145 (48.3%)	0.738
No formal education	15 (4.7%)	35 (11.7%)	<0.001
Admitted to a hospital within the last 6 months	102 (31.9%)	45 (15.0%)	<0.001
Was prescribed antibiotics within the last 6 months	201 (62.7%)	196 (65.3%)	0.652
Used antibiotics without a prescription within the last 6 months	220 (68.8%)	109 (36.3%)	<0.001

*^a^Calculated by x^2^ test or Mann–Whitney U test as appropriate.*

There were 458 CTX-M ESBL-producing Enterobacteriaceae (458/620, 73.9%) confirmed by PCR in this survey (**Table 2**), and the isolates were from different subjects. Compared with Ningyang county, Xintai county had a significant higher isolation rates of CTX-M-type ESBL-producing Enterobacteriaceae (*P*< 0.001). The genotyping results of *bla*_CTX-M_ gene demonstrated that 303 CTX-M-carrying isolates belonged to the CTX-M-9 group (303/458, 66.2%), followed by the CTX-M-1 group (146/458, 31.9%), and CTX-M-8 group (9/458, 1.9%). But there was no isolates belonging to the CTX-M-2 group. The dominant species among CTX-M ESBL-producing Enterobacteriaceae were *E. coli* (403/458, 88.0%) and *K. pneumoniae* (26/458, 5.7%). In addition, *Citrobacter* and *Enterobacter* were found among the CTX-M-type ESBL producers (**Table 3**).

**Table 2:** Detection of CTX-M-type extended-spectrum beta-lactamase (ESBL)-producing Enterobacteriaceae.

Locations	Participants	ESBL phenotype^a^		CTX-M gene^b^		CTX-M group^c^							
						CTX-M-1^d^		CTX-M-2^e^		CTX-M-8^f^		CTX-M-9^g^	
Xintai	320	271	84.7%	251	78.4%	88	35.1%	0	0.0%	6	2.4%	170	67.7%
Ningyang	300	241	80.3%	207	69.0%	58	28.0%	0	0.0%	3	1.4%	133	64.3%
Total	620	512	82.6%	458	73.9%	146	31.9%	0	0.0%	9	1.9%	303	66.2%

*^a^ESBL phenotype was determined according to CLSI recommendations.*

*^b^CTX-M gene was determined by PCR.*

*^c^Genotype of CTX-M genes was determined by PCR.*

*^d^CTX-M-1 group includes CTX-M-1 and several other variants, such as CTX-M-3 and CTX-M-15.*

*^e^CTX-M-2 group includes CTX-M-2 and several other variants.*

*^f^CTX-M-8 group includes CTX-M-8 and several other variants.*

*^g^CTX-M-9 group includes CTX-M-9 and several other variants, such as CTX-M-14.*

**Table 3:** Species composition of CTX-M-type ESBL-producing Enterobacterioceae.

Strains	No. (X/N)	Percentage (%)
*Escherichia coli*	403 (226/177)	88.0
*Klebsiella pneumoniae*	26 (19/7)	5.7
*Enterobacter cloacae*	12 (7/5)	2.6
*Enterobacter aerogenes*	10 (6/4)	2.2
*Citrobacter freundii*	7 (4/3)	1.5
Total	458	100.0

X, Xintai county; N, Ningyang county.

**FIGURE 1:**
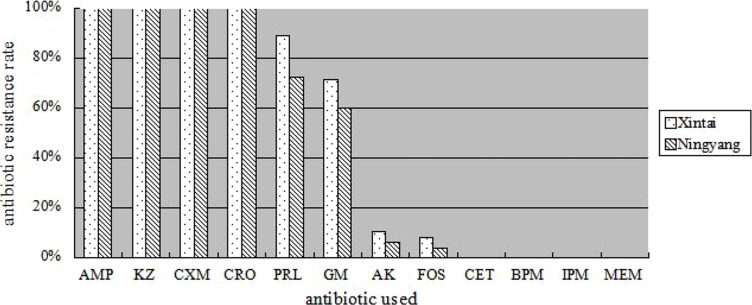
**Resistance rates of CTX-M extended-spectrum beta-lactamase (ESBL)-producers to 12 antibiotics.** AMP, ampicillin; KZ, cefazolin; CXM, cefuroxime; CRO, ceftriaxone; PRL, piperacillin; GM, gentamicin; AK, amikacin; FOS, fosfomycin; CET, cefoxitin; BPM, biapenem; IPM, imipenem; MEM, meropenem.

Among the CTX-M-type ESBL-producing isolates, they were all resistant to ampicillin, cefazolin, cefuroxime, and ceftriaxione, but were all susceptible to cefoxitin, biapenem, imipenem, and meropenem (**Figure 1**). The drug-resistance rate of CTX-M ESBL producers isolated from Xintai county to piperacillin, gentamicin, amikacin, and fosfomycin was significantly higher than from Ningyang (*P*= 0.013).

The multivariate logistic regression model used in this study identified the enrollment in formal education (OR 2.321; 95% CI 1.302–3.768; *P*= 0.039), the hospitalization history within the last 6 months (OR 1.753; 95% CI 1.127–2.584; *P*= 0.031), and the antibiotics use within the last 6 months (OR 1.892; 95% CI 1.242–2.903; *P*= 0.034). The three variables were significantly associated with carriage of CTX-M ESBL producers (*x*^2^ = 21.21; *df* = 3; *P*< 0.001).

## Discussion

Although there were several reports in China about the prevalence of ESBL-carrying Enterobacteriaceae in healthy people, the subjects were from city residents (Yu et al., 2007; Hu et al., 2013). Up to date, there is little information about isolation rate of ESBL-carrying Enterobacteriaceae in the rural residents in China, especially its risk factors associated with fecal carriage. The result of this present study demonstrated that high prevalence of CTX-M type ESBL-producing Enterobacteriaceae existed in the healthy rural residents in Taian, China (73.9%). The result showed a more or less similar prevalence with previous surveys in China and other country (Yu et al., 2007; Tian et al., 2008; Luvsansharav et al., 2012; Hu et al., 2013; Sun et al., 2014). Moreover, among CTX-M-positive isolates, the most dominant genotype was CTX-M-9 group (66.2%) and the dominant species were *E. coli* (88.0%). The results were in agreement with previous investigations about ESBL-producing Enterobacteriaceae isolated hospitals in China (Munday et al., 2004; Zhang et al., 2014).

The results of antimicrobial resistance testing revealed that all the CTX-M producers isolated from the rural residents were resistant to ampicillin, cefazolin, cefuroxime, and ceftriaxone, but all isolates were susceptible to biapenem, imipenem, and meropenem. The drug-resistant characteristics of CTX-M producers isolated from the rural residents are similar with those of ESBL-positive isolates from Chinese hospitals (Zhang et al., 2014).

The risk factor analyses in this study showed that the use of antibiotics and the hospitalization history within the past 6 months exhibited higher risks for carrying CTX-M ESBL-producing Enterobacteriaceae in this study. The result was consistent with the previous researches (Tian et al., 2008; Luvsansharav et al., 2012). Additionally, the rural residents in Xintai had a relatively better education and more frequent use of antibiotics, and they had a higher prevalence of CTX-M type ESBL-producing Enterobacteriaceae compared with humans from Ningyang. The result may be related the fact that humans with good economic income in the rural area of China are more likely to take some nonprescription drugs (Sun et al., 2014).

There were some limitations in this study. Fecal sampling was carried out only in two counties in Taian, so the results may not be representative of the whole area. The risk factor analysis was conducted according to the self-reporting of subjects, so recall bias such as the past drug use, may occurred. Most of the antibiotics tested were beta-lactams, but antibiotics of other classes were relatively less. Further sequence analysis of the CTX-M genes was not conducted.

In summary, the healthy rural residents in Xintai and Ningyang counties had high fecal carriage of CTX-M-type ESBL-producing Enterobacteriaceae. It is very important for the public health-care departments to educate the rural residents to rationally take over-the-counter drugs, control hospital infection strictly, and guide doctors to prescribe proper antibiotics for rational duration.

## Conflict of Interest Statement

The authors declare that the research was conducted in the absence of any commercial or financial relationships that could be construed as a potential conflict of interest.
